# The Potential of Stem Cell Therapy to Repair White Matter Injury in Preterm Infants: Lessons Learned From Experimental Models

**DOI:** 10.3389/fphys.2019.00540

**Published:** 2019-05-09

**Authors:** Josine E. G. Vaes, Marit A. Vink, Caroline G. M. de Theije, Freek E. Hoebeek, Manon J. N. L. Benders, Cora H. A. Nijboer

**Affiliations:** ^1^NIDOD Laboratory, University Medical Center Utrecht, Utrecht University, Utrecht, Netherlands; ^2^Department of Neonatology, University Medical Center Utrecht, Utrecht University, Utrecht, Netherlands

**Keywords:** preterm birth, white matter injury, mesenchymal stem cells, brain development, white matter pathology, cell therapies, regeneration, myelin loss

## Abstract

Diffuse white matter injury (dWMI) is a major cause of morbidity in the extremely preterm born infant leading to life-long neurological impairments, including deficits in cognitive, motor, sensory, psychological, and behavioral functioning. At present, no treatment options are clinically available to combat dWMI and therefore exploration of novel strategies is urgently needed. In recent years, the pathophysiology underlying dWMI has slowly started to be unraveled, pointing towards the disturbed maturation of oligodendrocytes (OLs) as a key mechanism. Immature OL precursor cells in the developing brain are believed to be highly sensitive to perinatal inflammation and cerebral oxygen fluctuations, leading to impaired OL differentiation and eventually myelination failure. OL lineage development under normal and pathological circumstances and the process of (re)myelination have been studied extensively over the years, often in the context of other adult and pediatric white matter pathologies such as stroke and multiple sclerosis (MS). Various studies have proposed stem cell-based therapeutic strategies to boost white matter regeneration as a potential strategy against a wide range of neurological diseases. In this review we will discuss experimental studies focusing on mesenchymal stem cell (MSC) therapy to reduce white matter injury (WMI) in multiple adult and neonatal neurological diseases. What lessons have been learned from these previous studies and how can we translate this knowledge to application of MSCs for the injured white matter in the preterm infant? A perspective on the current state of stem cell therapy will be given and we will discuss different important considerations of MSCs including cellular sources, timing of treatment and administration routes. Furthermore, we reflect on optimization strategies that could potentially reinforce stem cell therapy, including preconditioning and genetic engineering of stem cells or using cell-free stem cell products, to optimize cell-based strategy for vulnerable preterm infants in the near future.

## Introduction

Preterm birth is defined as birth before 37 weeks of gestation, and is relatively common with global prevalence rates ranging between 8 and 10%. Some of these children are born before 28 weeks of pregnancy (∼5% of all preterm births worldwide), and are labeled extremely preterm (Blencowe et al., 2012). White matter injury (WMI) is the most prevalent form of brain injury in the (extremely) preterm neonate and can lead to life-long neurological deficits (Back and Miller, 2014). While mortality rates following (extreme) preterm birth are steadily declining, the incidence of neurological sequelae remains high throughout the preterm population (Deng, 2010). It is estimated that about 25–50% of surviving extreme preterm infants encounter long-term neurological impairments, ranging from perceptual disabilities, impaired cognitive functioning, and behavioral problems, to an increased risk of psychiatric disorders (Larroque et al., 2008; Moster et al., 2008; Johnson et al., 2009; MacKay et al., 2010; Linsell et al., 2018). A smaller percentage of this population (5–10%) is believed to suffer from major motor problems, such as cerebral palsy (Larroque et al., 2008; Johnson et al., 2009).

Preterm WMI is thought to be the result of myelination failure during white matter development in the third trimester (Back et al., 2001; Khwaja and Volpe, 2008). The formation of myelin sheaths is essential for rapid, saltatory conduction of action potentials throughout the central nervous system (CNS), ensuring optimal brain connectivity, as well as protection of axonal integrity (Freeman and Rowitch, 2013). Even though the white matter is undeniably affected in preterm brain injury, evidence supporting brain injury in the preterm infant as a complex constellation of multiple neurodevelopmental disturbances, called “encephalopathy of prematurity,” has increased over the years (Volpe, 2009). These disturbances were shown to primarily involve the white matter, accompanied by (secondary) neuronal/axonal deficits affecting multiple brain regions, such as thalamus, basal ganglia, cerebral cortex, cerebellum, and brain stem (Volpe, 2009). Interestingly, recent studies have shed light on the development of another important cell type emerging in the third trimester, the (cortical) interneuron. Preterm birth was shown to affect both interneuron neurogenesis and migration, leading to disturbed interneuron distribution in the cortex in both a rabbit model of preterm WMI and post-mortem human tissue (Panda et al., 2018; Tibrewal et al., 2018). However, due to the irrefutable and fundamental role of impaired white matter development in preterm brain injury, this review focuses specifically on the protective and/or regenerative potential of treatments on the *white matter* of the brain.

The nomenclature in preterm WMI is one that can be hard to decipher. Before going into detail on the pathophysiology underlying preterm WMI it is important to clear up these terms to avoid confusion. Attempts to provide a consistent nomenclature have been made by combining neuroimaging findings with neuropathological correlates (Volpe, 2017). “Preterm WMI” is a collective name for a range of pathologies of the white matter in the developing brain. Based on neuropathological studies subdivisions into periventricular leukomalacia (PVL) and diffuse white matter injury (dWMI) can be made. PVL can be subdivided based on severity of necrosis and cyst formation. Punctate white matter lesions, sometimes recognized as a separate entity, are believed to result from small necrotic lesions and can be categorized in the PVL spectrum (Volpe et al., 2011; Back, 2017; Lee, 2017; Volpe, 2017; Zaghloul and Ahmed, 2017). dWMI is characterized by diffuse, subtle alterations in the white matter microenvironment without focal necrosis. Currently, preterm dWMI is the most prevalent form of WMI observed in preterm infants; it is believed that 80% of affected preterm neonates suffer from this type of WMI, leading to global hypomyelination (Back and Miller, 2014; Back, 2017). For this reason, we mainly focus on dWMI in this review.

Despite being a cause of serious neurological morbidity, treatment options for dWMI in preterm infants are still lacking. Although preterm dWMI differs from other (adult) CNS disorders in etiology and symptoms, the majority of these other conditions are (in part) caused by damage to the white matter and/or insufficient (re)myelination, resulting in abnormal brain functioning. Therefore, research already performed from these other areas of white matter pathology could aid in the identification and optimization of potent treatment strategies to combat preterm dWMI. Here we will discuss the potency of stem cell-based treatments for dWMI, by reviewing a wide range of *in vitro* and *in vivo* studies in multiple adult and pediatric white matter diseases.

## Preterm White Matter Injury: Pathophysiology

Preterm infants are born at a very crucial period of cerebral white matter development, since myelination starts only around 32 weeks of gestation (Back et al., 2001; Knuesel et al., 2014). Prior to this gestational age, the myelin-forming cells of the brain, i.e., oligodendrocytes (OLs), undergo highly regulated and strictly timed developmental changes in order to transform into mature OLs capable of myelin production. OLs typically develop via a 4-stage program: (1) neural stem cells (NSCs) originating from different endogenous stem cell niches of the brain [for example the lateral subventricular zone (SVZ)] develop into, (2) OL precursor cells (OPCs), which migrate to designated brain regions. There, the OPC population will expand through proliferation and subsequently differentiates into, (3) immature pre-myelinating OLs (pre-OLs) that progress to the final stage of 4) mature myelinating OLs (Back et al., 2001; Emery, 2010; Volpe et al., 2011; van Tilborg et al., 2018). OPCs remain present in the brain throughout adulthood and are crucial for myelin maintenance and remyelination of axons after damage. Any disturbance in local OPC pools by differentiation, migration or cell death will be rapidly restored via multiple pathways that regulate OPC proliferation, ensuring a homeostatic number of OPCs (Bradl and Lassmann, 2010; van Tilborg et al., 2016). OL lineage maturation and migration in the developing brain has been described in detail in multiple excellent studies (Kessaris et al., 2006; Jakovcevski et al., 2009; Mitew et al., 2014; van Tilborg et al., 2018).

The majority of OL lineage cells present in the brain of infants born between 24 and 32 weeks are OPCs and pre-OLs (Back et al., 2001, 2007; Volpe et al., 2011). These immature cell types have been reported to be very sensitive to preterm birth-related insults, while mature OLs are more resilient to damage (van Tilborg et al., 2016; Bennet et al., 2018). Accumulating evidence has identified inflammation and hypoxia, both insults unequivocally linked to preterm birth, as two main pathways involved in disruption of OL lineage development (Khwaja and Volpe, 2008; Volpe, 2009; Deng, 2010). These two detrimental types of insults are believed to work synergistically, as the incidence of WMI has shown to be higher in children exposed to multiple insults (Rezaie and Dean, 2002; Zhao et al., 2013). A pro-inflammatory state of the brain can result as a consequence of antenatal sequelae like maternal inflammation and/or intra-amniotic infections (often a trigger of preterm birth), or due to postnatal infections such as neonatal sepsis (Back and Miller, 2014; van Tilborg et al., 2016; Bennet et al., 2018). Irrespective of timing, inflammation is thought to contribute to WMI through systemic cytokine release and the activation of microglia (i.e., microgliosis), the innate immune cells of the brain. As a consequence of microgliosis, toxic compounds such as free radicals, glutamate and pro-inflammatory cytokines, including tumor necrosis factor-α (TNF-α), interleukin (IL)-17, interferon-γ (IFN-γ), and IL-1β, are secreted in the brain leading to pre-OL injury (Favrais et al., 2011; Hagberg et al., 2012; van Tilborg et al., 2016). In addition to inflammation being an important hit for WMI, preterm birth is also linked to disrupted cerebral oxygen levels in the perinatal period as preterm infants often need mechanical ventilation because of an underdeveloped respiratory system (Brown and DiBlasi, 2011). Furthermore, preterm infants have an underdeveloped cardiovascular system with disturbed autoregulation of cerebral blood flow (Fyfe et al., 2014). Taken together, these events can ultimately lead to low blood pressure, hypocapnia, leading to cerebral vasoconstriction, and brain hypoperfusion (Viscardi et al., 2004; Fyfe et al., 2014). Apart from the risk of hypoxia, oxygen disturbances in preterm infants are possibly also caused by periods of hyperoxia following excessive ventilation, all the while optimal oxygen saturation levels in preterm infants are still under debate (Lakshminrusimha et al., 2014; Stoll et al., 2015). Whereas mature OLs largely tolerate hypoxic insults, pre-OLs are very vulnerable to an imbalanced oxygen supply. In addition, disruptions of the oxygen supply leads to oxidative stress through various pathways, ultimately causing production and accumulation of reactive oxygen species within developing OLs (van Tilborg et al., 2016). For example, activation of nitric oxide synthase causes pre-OL injury by nitric oxide production (Gluckman et al., 1992; Khwaja and Volpe, 2008; Lee, 2017; Singh et al., 2018). Further, OPCs are extremely sensitive to oxidative damage, as they lack particular anti-oxidant enzymes (Perrone et al., 2015). Moreover, hypoxia has been shown to further fuel activation of the immune system, including activation of microglia thereby augmenting the release of pro-inflammatory cytokines (Singh et al., 2018).

Even though myelination failure is evident in preterm dWMI, the exact pathophysiology of the OL lineage has yet to be determined. On the one hand it is hypothesized and supported by human post-mortem and experimental animal studies that an initial wave of pre-OL cell death is compensated by an inadequate regenerative response from the large reservoir of early OL progenitors, leading to a secondary OL maturational arrest in these newly formed cells (Robinson et al., 2006; Segovia et al., 2008; Back, 2015). On the other hand, several human post-mortem studies failed to show any evidence of OL lineage cell death in dWMI, which suggests that arrested maturation of the large pool of pre-existing OPCs and pre-OLs underlies dysregulated myelination observed in preterm dWMI (Billiards et al., 2008; Buser et al., 2012; Verney et al., 2012).

Regardless of the exact nature of myelination failure in preterm dWMI, ultimately, the lack of proper myelination during brain development will negatively influence axonal processes leading to impaired connectivity and causing life-long neurodevelopmental deficits (van Tilborg et al., 2016).

## Stem Cell Therapy in Adult and Pediatric White Matter Pathologies

Although dWMI can cause long term neurological impairments, there are currently no treatment options available to reduce myelination deficits in the developing brain. A prospective therapy for preterm WMI would preferably be multifaceted, and thus act on multiple pathophysiological processes contributing to preterm WMI. Displaying both anti-inflammatory properties as well as providing trophic support, mesenchymal stem cells (MSCs) have been proposed as a potent therapeutic tool in numerous neuropathologies, including white matter diseases (Kassis et al., 2008; Liang et al., 2014). MSCs are believed to exert their regenerative abilities by adaptation of their secretome *in situ*, favoring endogenous repair of brain injury through paracrine signaling (van Velthoven et al., 2010a; Kassis et al., 2011; Liang et al., 2014; Paton et al., 2017). MSCs can be harvested from a wide range of tissues, including bone marrow, adipose tissue, and the umbilical cord (both from the Wharton’s jelly and from cord blood) (Kobolak et al., 2016; Volkman and Offen, 2017). Moreover, MSC administration has a low risk of triggering the recipient’s immune system, due to low expression of major histocompatibility complex (MHC) class I receptors, lack of MHC class II receptors and lack of co-stimulatory proteins (e.g., CD40, CD80, and CD86) on the MSC’s plasma membrane (De Miguel et al., 2012; Jacobs et al., 2013). While MSC therapy could be an attractive therapeutic option, evidence supporting the regenerative effect of MSCs in dWMI is still scarce. Research on MSCs used in *in vitro* models or in other brain pathologies could contribute to more insight into development of an effective cell-based therapy for the vulnerable white matter of the preterm brain. Therefore we start by discussing data obtained in studies using MSC therapy in basic *in vitro* models of OL development and in *in vivo* models of adult and neonatal conditions with pronounced WMI.

### Mesenchymal Stem Cells in *in vitro* Models of OL Development

Evidence of a potential direct effect of MSCs on OL development in *in vitro* models of WMI is scarce. A few studies report a supportive role of the MSC secretome in OL differentiation. Zhang et al. (2016) studied the direct effect of rat ectodermal MSCs (derived from the neural crest) in both a non-contact (transwell) and cell-cell contact co-culture with OPCs. Interestingly, both non-contact and direct contact MSC co-cultures significantly improved the number of myelin basic protein (MBP), a structural component of mature myelin exclusively expressed by mature myelinating OLs, positive (mature) OLs and the length of the OL processes (important for sufficient axonal wrapping) compared to OPCs cultured without the presence of MSCs, indicating at least partially an important role of the MSCs’ secretome. However, the most pronounced increase in mature OL numbers and process outgrowth was found in the direct contact co-culture, indicating an additional positive effect through direct cell-cell contact or near-proximity of the MSCs. A study adopting a similar setup, but using rat NSCs instead of OPCs, reported comparable results. Direct co-culture of NSCs and human Wharton’s jelly derived MSCs (WJ-MSCs) led to a greater increase in the expression of MBP and the immature OL marker GalC, compared to exposure of NSCs to only the MSCs’ secretome via either non-contact WJ-MSC-NSC co-cultures or by using WJ-MSC conditioned medium (Oppliger et al., 2017). Direct MSC contact is believed to lead to superior OL maturation and process outgrowth through the presence of gap junctions and extra-cellular matrix (ECM) proteins, such as laminin, produced by MSCs (Zhang et al., 2016; Oppliger et al., 2017). Multiple other studies have shown that MSCs are capable of mitochondrial transfer through microvesicles, gap junctions or nanotubes to cells with impaired mitochondrial function following oxidative stress, a detrimental hit to developing OLs (Liang et al., 2014; Lin et al., 2015; Mahrouf-Yorgov et al., 2017; Paliwal et al., 2018). Even though studies demonstrating mitochondrial transfer between MSCs and damaged OLs are currently lacking, this mechanism could contribute to the observed superior effect of cell proximity. The additive effect of cell proximity is not supported in all available *in vitro* studies. For instance, Rivera et al. (2006) demonstrated comparable effects of either direct cell-cell contact of MSCs or only using MSC-conditioned medium (CM) in the promotion of oligodendrogenesis in a rat NSC culture.

Thus, based on these findings it seems that MSCs and their secretome play a supportive role in OL maturation, even though a close proximity of the two cell types might be of importance for the most optimal effect. The final location of stem cells in the brain and their proximity to target cells could be further elucidated by studying the biodistribution, migration and cellular niches of transplanted MSCs in *in vivo* models of preterm dWMI for instance by using fluorescent labeled stem cells, bioluminescence or tracing of xenogenic transplants. It is, however, relevant to note that in none of the above described *in vitro* studies OPCs or NSCs were challenged, meaning that the effect of MSCs on oligodendrogenesis was studied under non-injured circumstances. What the effects of MSCs could be on maturation when OPCs are challenged with inflammatory or injury-mimicking stimuli is yet to be studied. Moreover, most *in vitro* studies focus on the effect of MSC on NSCs and endogenous regeneration of myelination through formation of new OL progenitors in the stem cell niche of the SVZ. However, the primary therapeutic target in dWMI might be the maturation-arrested pre-OLs residing in the injured white matter, so additional research needs to be done to study the potential beneficial effects of MSCs in *in vitro* models mimicking maturational arrest of OL progenitors.

### Mesenchymal Stem Cells in Adult White Matter Disease

To assess the potency of MSCs in preterm WMI, this section focuses on adult pathologies in which the *white matter* of the brain is affected. The potency of MSCs in other adult brain diseases in which primarily the gray matter is affected can be found elsewhere (Laroni et al., 2013; Volkman and Offen, 2017).

#### Stroke

The effectiveness of MSC therapy to repair the brain following stroke, a pathological condition in which disturbances in cerebral blood flow lead to permanent neurological impairments, has been studied extensively over the past years. A recent meta-analysis by Sarmah et al. (2018) reported significant improvement of neurological deficits following ischemic stroke compared to controls in all included animal studies. Even though the majority of these studies mainly address the effects of MSC therapy on regeneration of the gray matter, the white matter is also damaged in cerebrovascular disease but fewer studies have specifically focused on the effects of MSC treatment on the damaged white matter after stroke (Jiang et al., 2006; Gutierrez-Fernandez et al., 2013b; Mifsud et al., 2014; Hayakawa and Lo, 2016). Yu et al. (2018) showed a significant increase in MBP levels, the key protein in myelin sheaths, after intraventricular rat bone marrow-derived MSC (BM-MSC) administration following transient middle cerebral artery occlusion (MCAO) in rats. The increase in MBP expression was hypothesized to be the result of the reported rise in proliferating OPCs following BM-MSC treatment. Moreover, fractional anisotropy (FA) values, a measure of WM integrity determined by MRI-DTI, were lower in animals that did not receive cell therapy. In addition, Gutierrez-Fernandez et al. (2013a) showed an incline in Olig2 protein levels, an OL marker that marks all developmental stages, after intravenous rat BM-MSC and rat adipose tissue-derived MSC (AD-MSC) treatment in a rat model of focal ischemia induced by permanent MCAO (Gutierrez-Fernandez et al., 2013a). Furthermore, in a rat model of subcortical stroke, intravenous rat AD-MSC administration increased the number of OL progenitors in the area of stroke and the number of mature OLs in the penumbra, leading to an increase in myelin formation and hence restoration of white matter integrity (Otero-Ortega et al., 2015). Thus, these *in vivo* studies show potent reduction in myelination deficits following MSC therapy after stroke, which could be related to the observed increase in OL progenitor proliferation. Moreover, they also underline the proposed transient *paracrine* effects of MSCs as the surviving number of engrafted or differentiated transplanted cells is very small in models of stroke/MCAO (Dulamea, 2015; Cunningham et al., 2018; Sarmah et al., 2018).

Even though brain ischemia is responsible for the greater percentage of all strokes, the pathological term “stroke” does not only entail ischemic cerebrovascular accidents. *Hemorrhagic* stroke, including intracerebral hemorrhage (ICH), accounts for about 10–20% of strokes. However, studies investigating the potential of MSC treatment in experimental models of hemorrhagic stroke are less prevalent. A recent review by Bedini et al. (2018) did report enhanced functional recovery and reduction in lesion size in animal models of ICH as a result of MSC treatment. In a rat model of striatal ICH, intraventricular injection of human WJ-MSCs, led to a decrease in myelination deficits as shown by luxol fast blue staining (which stains phospholipids in myelin) and upregulation of MBP protein levels confirmed by Western Blot, suggestive of an increase in remyelination (Liu et al., 2010). Intranasal rat BM-MSC therapy in a rat model of subarachnoid hemorrhage (SAH), in which brain injury is evoked by presence of blood in the subarachnoid space and subsequent cerebral ischemia, was recently shown to reduce white matter loss, demonstrated by a rise in MBP expression (Nijboer et al., 2018).

The encouraging results in preclinical studies have prompted various clinical trials to assess the safety, feasibility and efficacy of MSC treatment in stroke patients. A meta-analysis analyzing a large number of the clinical studies in Asia confirmed the safety and efficacy of MSC therapy in ischemic stroke (Xue et al., 2018). Neurological deficits were significantly reduced, while no serious adverse events were reported. Interestingly, outcome parameters did not differ significantly between patient groups treated in the acute phase or chronic phase of ischemic stroke, indicating a wide treatment window using MSCs. Other sub-analyses for optimal dosage, cell origin and administration methods were not conclusive (Xue et al., 2018). Toyoshima et al. (2017), who reviewed a different subset of clinical studies, reported similar findings while stressing the need for additional research to determine optimal timing, route of administration and dosages.

#### Multiple Sclerosis

Multiple sclerosis (MS), a disorder in which a dysregulated autoimmune response is believed to result in transient and eventually chronic demyelination of the CNS, is one of the leading causes of neurological deficits in young adults (Compston and Coles, 2002; Uccelli et al., 2007). The exact pathophysiological mechanism of disease onset and progression is beyond the scope of this review and multiple excellent reviews on this subject can be found elsewhere (Ciccarelli et al., 2014; Garg and Smith, 2015; Correale et al., 2017; Thompson et al., 2018). Due to the persistent and uncontrolled T-cell, B-cell and microglial activation, a prospective therapy for MS should both attenuate the autoimmune attack, and promote remyelination/axonal regeneration (Uccelli et al., 2007). Over the years, animal studies have explored the potential of MSC therapy in MS and the results have been summarized in detail in many other reviews (Jadasz et al., 2012; Morando et al., 2012; Rivera and Aigner, 2012; Cohen, 2013; Laroni et al., 2013; Gharibi et al., 2015; Xiao et al., 2015; Genc et al., 2018). These studies frequently use animal models of either toxin-induced (i.e., cuprizone) demyelination or an experimental autoimmune encephalitic (EAE) model, mimicking inflammation-induced demyelination by active immunization with myelin- or OL associated antigens (Jadasz et al., 2012). Genetic models, like *Shiverer* mice, in which an autosomal recessive mutation leads to CNS hypomyelination, are also used to study MS. One of these studies, by Cristofanilli et al. (2011), used an unconventional approach by co-transplanting mouse BM-MSCs with allogenic OPCs intracranially to boost remyelination in demyelinated *Shiverer* mutants. They hypothesized that BM-MSCs would display immunosuppressive properties, boosting allogenic OPC engraftment. The co-transplantation resulted in an increase in myelination surrounding the injection site and was due to both reduction in inflammation and a boost of OPC engraftment, migration and differentiation. While BM-MSC therapy alone was shown to dampen the inflammatory response, a direct comparison between BM-MSC and BM-MSC plus OPC therapy on other outcome parameters was not made. Therefore it is unclear if the regenerative effect was the result of combination therapy, or could also be achieved by BM-MSC therapy alone. However, this study highlights the potent anti-inflammatory effect that MSCs can have in the injured white matter. Even though these preclinical studies all report improvement of histological and behavioral outcomes after MSC therapy (either applied intravenously, intracerebrally, or intraperitoneally) in models of relapse-remitting or chronic MS, the mechanism of action of MSCs in these models is unclear. Many of these studies report reduction in demyelination as a result of modulation of the immune system, reducing peripheral T-cell and B-cell influx or activation (Zappia et al., 2005; Zhang et al., 2005; Kassis et al., 2008; Bai et al., 2009; Liu et al., 2009; Cristofanilli et al., 2011; Liu et al., 2013). In contrast, the regenerative role of MSCs following white matter damage seems less pronounced in models of MS. However, some of the EAE studies do report an increase in endogenous oligodendrogenesis following MSC therapy, as a result of the MSCs’ secretome (Zhang et al., 2005; Kassis et al., 2008; Bai et al., 2009; Jaramillo-Merchan et al., 2013; Liu et al., 2013). Nessler et al. (2013) used a mouse model of cuprizone-induced CNS demyelination, to assess the potency of MSCs to repair myelination deficits without interference of immune system activation. Interestingly, neither intranasal nor intravenous application of human or mouse BM-MSCs were shown to beneficially affect myelination. Another study in the same model performed by Cruz-Martinez et al. (2016) showed opposite results: intraventricular injection with mouse BM-MSCs led to increased OL progenitor proliferation in the SVZ and myelin regeneration at the lesion site. The contrast in outcome of these studies could be related to differences in administration routes of MSCs, as Nessler et al. (2013) concluded that the lack of myelin regeneration following MSC therapy was related to the intact blood brain barrier (BBB), limiting MSC migration toward the lesion site following intravenous or intranasal administration. Cruz-Martinez et al. (2016) chose a direct approach by injecting the MSCs in the lateral ventricles of the brain. In conclusion, data from these studies indicate that neuroinflammation with a strong chemotactic signal and damaged BBB facilitates MSC migration and could be the key for MSC-based therapies to effectively remyelinate the damaged white matter.

Taken together, available data indicate that MSCs have beneficial effects in animal models of MS through either their anti-inflammatory properties or regenerative properties. Over the years, multiple small studies exploring safety and feasibility of MSCs therapy in MS patients have been published. A recent clinical review by Scolding et al. (2017) provides a clear overview of the outcome of these studies. In general, MSC therapy was warranted to be safe, with very little reported adverse events, including a study ruling out neoplasia formation (von Bahr et al., 2012; Scolding et al., 2017). Currently, several phase II trials using MSCs for MS are underway (SIAMMS-II; NCT01932593, ACTiMuS; NCT01815632, and MESEMS; NCT01854957).

In conclusion, in adult experimental models for stroke and MS it has been shown that MSCs can have both regenerative and anti-inflammatory paracrine effects by which white matter deficits can be restored. The exact working mechanism of MSCs in different pathologies, however, seems to be dependent on the underlying pathophysiology of the disease and the administration route.

### Mesenchymal Stem Cells in Term Neonatal Brain Pathologies

#### Neonatal Hypoxic-Ischemia Encephalopathy

Aside from the abundance of studies showing the potency of MSC therapy in adult white matter disease, the evidence supporting MSC treatment in neonatal brain injury has grown steadily over the years. A vast amount of preclinical research has focused on a relatively prevalent form of neonatal brain injury, hypoxic-ischemic encephalopathy (HIE) in the term infant.

**FIGURE 1:**
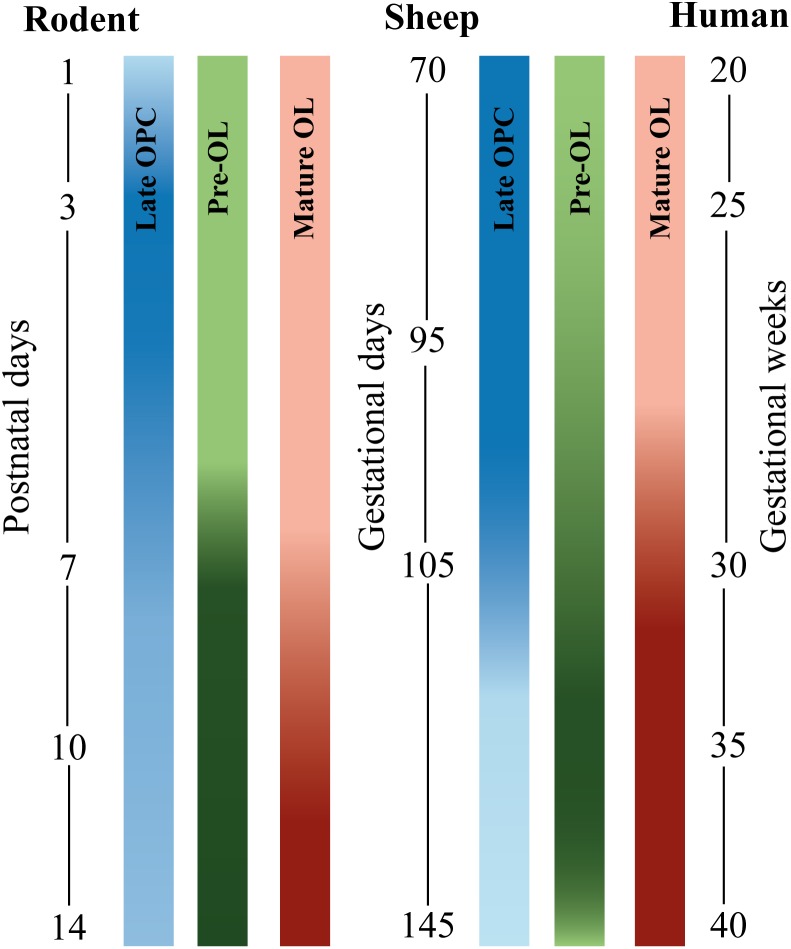
Developmental timeline comparing oligodendrocyte (OL) stage-specific development in different species. Blue bars depict late OL precursor cells (OPC), green bars depict pre-myelinating precursors (pre-OLs), and red bars depict mature OLs. From left to right: rodent, sheep, and human. The postnatal window in rodent OL development between postnatal day (P1–P14) corresponds to the latter half of human gestation [data are based on (Craig et al., 2003) and (Salmaso et al., 2014)]. Fetal sheep OL development between 70 and 145 gestational days (GD) approximately corresponds with late second and third human trimester [data based on (Back et al., 2012)]. Human OL development is based on data from Back et al. (2001). The intensity of the bar indicates the peak of OL development. Note that OL development of human extreme preterms (24–28 weeks of gestation) roughly corresponds to rodent P2 to P5 and ovine 90–95 GD. Also note that sheep and human OL development are roughly comparable, whereas rodent OL development is slightly different regarding time-window of OL development and composition of OL subtypes per postnatal/gestational age (Craig et al., 2003).

Hypoxic-ischemic encephalopathy can be the result of perinatal asphyxia, in which a birth-related event, such as shoulder dystocia or collapse of the umbilical cord, leads to inadequate cerebral blood flow and oxygenation (Douglas-Escobar and Weiss, 2015). The decreased cerebral perfusion sets in motion a temporal sequence of detrimental insults, eventually leading to neuronal cell death in the cerebral cortex or basal ganglia and thalami (Douglas-Escobar and Weiss, 2015). In addition to gray matter injury, the white matter is also affected in HIE (Silbereis et al., 2010). Previous work in a 9-day-old (postnatal day 9, P9; for human gestation equivalence, see Figure 1) mouse model of HIE performed at our center showed that MSC therapy improved functional outcome and reduced lesion size following HIE by stimulating endogenous repair of the brain. Intracranial mouse BM-MSC transplantation was shown to boost neurogenesis, OL formation and reduced white matter loss (van Velthoven et al., 2010a). Further reduction in myelin loss was achieved with a second intracranial dose of MSCs, but this second MSC dose did not further increase oligodendrogenesis (van Velthoven et al., 2010c). The effect of MSC therapy on white matter integrity in the mouse HIE model was further investigated using DTI, showing normalization of FA values in the cortex and corpus callosum in MSC-treated animals. These results were confirmed by restored histological MBP intensity and pattern in similar brain areas (van Velthoven et al., 2012b). Other research by van Velthoven et al. (2011) provided evidence underlining the adaptive potential of the MSCs’ secretome, reporting multiple gene expression changes in growth factors and cytokines in MSCs, which are believed to be pivotal for cerebral cell survival, proliferation and differentiation, in response to the HIE milieu. Moreover, it was shown that MSCs are unlikely to integrate into the brain, as <1% of the cells could be detected 18 days after the last MSC administration (van Velthoven et al., 2011). In contrast to these findings, Park et al. (2013) reported that human AD-derived MSCs differentiated into MBP-expressing OLs following intracranial transplantation in their rat model of inflammatory HIE. In addition, the MSCs were shown to aid endogenous preservation of myelin by producing trophic factors and decreasing pro-inflammatory cytokines (Park et al., 2013). It is, however, important to note the differences between these two preclinical HIE models. The rat model of Park et al. (2013) displayed severe cystic WMI while the mouse model of van Velthoven et al. (2010a) displayed moderate neuronal loss and more global myelin deficits. A different study using a near-term (P7) mouse model of HIE, displaying demyelination, neuronal and OL loss, alterations in OL development and axonal damage, showed a positive effect of intraventricular human amniotic fluid stem cells (AFSCs) administration (directly following hypoxia) with a marked reduction in MBP loss after treatment. AFSCs were shown to express an important MSC marker, CD73. However, the protective effect was only observed in AFSCs with a spindle-shaped cytoplasm, while AFSCs with a rod-shaped cytoplasm were not capable to prevent myelination deficits (Corcelli et al., 2018). In addition to improvement of functional outcome or lesion size, multiple studies reported a reduction in cerebral inflammation following MSC therapy, confirming the immunomodulatory properties of MSCs (van Velthoven et al., 2010a; Donega et al., 2014b; Gu et al., 2015, 2016; Ding et al., 2017). While most studies focus on short-term outcome of MSC therapy in HIE, our study using a 14 months post-HIE follow-up found long lasting improvements of functional outcome and myelination in intranasally mouse BM-MSC-treated mice compared to vehicle-treated littermates. Moreover, pathological analysis of multiple organs did not reveal an increase in neoplasia following MSC treatment after this long-term follow up, indicating that intranasal MSC treatment was safe (Donega et al., 2015).

Based on these promising results, more recent studies have focused on the efficacy of MSC therapy in combination with clinical hypothermia, the only recommended clinical intervention in HIE to date. Herz et al. (2018) hypothesized that hypothermia immediately after HIE induction (32°C during 4 h) and subsequent intranasal mouse BM-MSC treatment 3 days later would lead to augmented neuroprotection and improvement of neurological outcome in P9 mice. However, while both single therapies improved behavioral outcome and MBP protein levels, combination therapy abolished these protective effects. Additional *in vitro* and *in vivo* experiments revealed that hypothermia might alter the microenvironment in the brain, negatively impacting the potential of the MSC secretome (Herz et al., 2018). In contrast, another recent report showed positive effects of combination therapy in a rat model of HIE (Park et al., 2015; Ahn et al., 2018a). In their P7 rat model of HIE the combination of hypothermia, started 6 h after HIE induction (32°C during 24 h), plus intraventricular human umbilical cord blood-derived MSCs (UCB-MSCs; MSCs selected from the cord blood) at the time of hypothermia induction, improved functional recovery and attenuation of inflammation, measured by a reduction in optical density of the macrophage lineage marker ED-1 and cerebrospinal fluid (CSF) concentrations of pro-inflammatory cytokines, compared to either therapy alone (Park et al., 2015). Moreover, in a subsequent study these authors reported a broader therapeutic window of UCB-MSCs, as MSC treatment directly after a 48 h period of hypothermia (32°C, started 3 h after the HI insult) attenuated HIE associated brain injury compared to MSC treatment without prior hypothermia (Ahn et al., 2018a). It is, however, important to note that these studies exhibit large methodological differences in their HIE model, mode of administration and cell source. Most importantly, while Herz et al. (2018) treated animals 3 days after hypothermia, both other studies administer MSCs during or directly after cooling of the animals. Even though the additive effect of MSC therapy following therapeutic hypothermia in HIE is still up for discussion, multiple phase I/II clinical trials are currently underway or have recently been completed (NCT01962233, NCT02434965, NCT02881970, NCT02612155, and NCT03635450). A pioneer trial on human umbilical cord blood cell (UCBC; contains MSCs but also other cells from cord blood) treatment following therapeutic hypothermia in neonates with HIE showed that intravenous autologous UCBC treatment within 72 h postnatal is feasible and was not associated with any significant short-term adverse events in a small group of patients (Cotten et al., 2014). However, it is important to note that umbilical cord blood can contain a variety of cells, including MSCs (Pimentel-Coelho et al., 2010).

#### Perinatal Arterial Ischemic Stroke

Another important type of ischemic brain injury in term neonates is perinatal arterial ischemic stroke (PAIS), a cerebrovascular accident predominantly involving the middle cerebral artery (MCA), which is associated with serious morbidity in term neonates (Kirton and deVeber, 2009). Even though PAIS differs in pathology and symptoms from stroke in adults, white matter deficits are present in both conditions. While the body of evidence supporting MSC therapy in PAIS is less profound compared to its adult counterpart and compared to neonatal HIE, there are some studies providing evidence for the therapeutic potential of MSCs in PAIS. A recent review from our group summarized the findings of all available studies focusing on the potential of MSC therapy in *in vivo* experimental models of PAIS (Wagenaar et al., 2018). While the amount of evidence is limited, some studies that mimic PAIS by (transient) MCA occlusion do report attenuation of WM loss following intranasal rat BM-MSC therapy on either MRI-DTI parameters or by using histology (van Velthoven et al., 2013, 2017). A clinical trial studying the safety and feasibility of intranasal allogenic BM-MSC administration in PAIS patients will start at our center in the near future (PASSIoN; NCT03356821).

### Mesenchymal Stem Cells in Preterm Brain Injury

The evidence provided supports MSCs in their ability to protect and regenerate the white matter after numerous types of injury in both the adult and neonatal brain. MSCs are shown to effectively stimulate OL survival, maturation and subsequent (re)myelination. Treatment options to combat preterm dWMI are currently lacking, as treatment possibilities such as hypothermia used in term HIE patients have shown to be ineffective or inapplicable in the preterm population (Deng, 2010). Nevertheless, preclinical studies investigating the potential of MSC treatment in specifically the diffuse form of preterm WMI are still limited. However, stem cell therapy for other pathologies in the preterm brain, such as intraventricular hemorrhage (IVH) and cystic PVL, have received some attention over the years.

#### Intraventricular Hemorrhage

Apart from being susceptible to impaired WM maturation, preterm neonates are also prone to develop an IVH. Severe IVH (grade III/IV), in which the germinal matrix hemorrhage breaks through the ependymal lining into the ventricular system, followed by post-hemorrhagic ventricular dilatation (PHVD) or even secondary venous infarction, can result in serious neurological morbidity (Brouwer et al., 2012; Payne et al., 2013; Park et al., 2017). While the exact pathophysiology remains unclear, severe IVH is associated with damage to the (periventricular) white matter and cortical neuron dysfunction (Park et al., 2017). Pioneering studies of intraventricular human UCB-MSC therapy in a rat model of severe IVH revealed attenuation of the inflammatory response, reduction in apoptosis, restoration of corpus callosum thickness and improvement of myelination following MSC therapy (Ahn et al., 2013). Moreover, the incidence of PVHD, an important cause of (secondary) injury to the periventricular white matter, was significantly reduced after MSC treatment. A follow-up study on the optimal route of human UCB-MSC administration showed that both intracranial and intravenous administration were equally effective to reduce inflammation, reduce corpus callosum thinning and boost myelination following severe IVH (Ahn et al., 2015). The effectiveness of intravenous administration of human WJ-MSCs was confirmed by Mukai et al. (2017), who reported attenuation of hypomyelination and periventricular apoptosis following MSC therapy in a mouse model of severe IVH. Additional studies on the optimal timing of stem cell therapy showed a relatively limited window of treatment, as intraventricular human UCB-MSC therapy was shown solely to be effective when administered at 2 days after IVH compared to a 7 day interval (Park et al., 2016). The beneficial effect of UCB-MSCs in severe IVH was shown to be in part mediated by MSC-secreted brain-derived neurotrophic factor (BDNF) (Ahn et al., 2017). These promising results initiated the first clinical trial on MSC therapy in severe IVH in preterm infants, in which both a low and high dose of intraventricular UCB-MSCs were found to be safe and feasible (Ahn et al., 2018b). To evaluate the therapeutic potential a phase II trial is currently being executed (NCT02890953).

#### Cystic PVL

An early study of MSC therapy for preterm WMI by Chen et al. (2010) used a rat model of cystic PVL. In this model, bilateral injection of excitotoxic ibotenic acid (IBA) into the white matter of P5 rats leads to myelin loss, along with transient cyst formation, microglia activation, and cerebral palsy-like behavioral deficits (Chen et al., 2008). Animals were treated with neonatal rat BM-MSCs by unilateral intracerebral injection at 1 day post-PVL. Important to note with this study was that control PVL animals received an injection with cell-free MSC-CM. MSCs were shown to migrate to both lesioned hemispheres, increased endogenous glial cell proliferation and led to improved myelination and motor outcome compared to control PVL rats. Even though injection with MSCs was more effective than cell-free MSC-CM administration, conclusions on the possible (limited) effects of MSC-CM could not be made due to the lack of a suitable vehicle-treated control group (Chen et al., 2010). Similar results were obtained by Zhu et al. (2014) who induced PVL by ligation of the left common carotid artery, followed by 4 h of hypoxia (6% O_2_) in P3 rats. Directly following PVL induction, rats received a daily intraperitoneal injection with human WJ-MSCs for 3 consecutive days. MSC treatment improved functional outcome in an open field test, reduced microglia and astrocyte activity and raised the amount of MBP-positive staining in the white matter. A more recent study induced PVL-like injury by an intraperitoneal lipopolysaccharide (LPS) injection (15 mg/kg) in P4 rats and demonstrated that intraperitoneal human WJ-MSC treatment significantly reduced pro-inflammatory cytokine expression in the brain and reversed the LPS-induced decrease in MBP-positive area (Morioka et al., 2017).

#### Diffuse WMI

While the studies on cystic PVL support the regenerative and anti-inflammatory capacities of MSCs, as discussed the most common form of preterm WMI is not focal necrosis but diffuse (non-cystic) WMI (Back, 2017). The effect of human WJ-MSC in diffuse WMI was studied by Mueller et al. (2017). In this study an intraperitoneal LPS injection (0.1 mg/kg) in P3 rat pups was followed by ligation of the left carotid artery combined with 40 min of hypoxia (8% O_2_) the next day (P4). At P11 the animals received intracranial WJ-MSC treatment. WJ-MSC transplantation led to improvement in locomotor activity and less myelin loss and astrocyte activation. While these results are promising, intracranial MSC administration lacks clinical applicability. A recent study investigated intranasal delivery of human WJ-MSCs in a rat model of dWMI induced by intraperitoneal injection of LPS (0.1 mg/kg) at P2 and left carotid artery ligation and hypoxia at P3 (Oppliger et al., 2016). Neonatal rats treated intranasally with MSCs showed a reduction in myelination deficits and gliosis compared to vehicle-treated dWMI littermates. This model was associated with pre-OL depletion that could not be reversed by MSC therapy. Interestingly, the authors were able to identify two phenotypes of mature OLs. In vehicle-treated dWMI animals they found an increase in OLs with a MBP-positive and Ki67-negative perikaryon (mature non-proliferating OLs), with weak MBP-positive extensions. In MSC-treated dWMI animals, many MBP-positive and Ki67-negative OLs were also shown, but in contrast, these cells showed bright, thick and elaborate MBP-positive cell processes, indicating myelination. Based on these observations, the authors hypothesized that the local cerebral environment resulting from the insult could hinder newly generated OLs to fully regain their function and proceed with remyelination. In line with that hypothesis, human WJ-MSCs could beneficially change this negative cerebral environment by secreting immunomodulatory or trophic factors, leading to proper maturation of OLs and subsequent increase in myelin production.

In addition to rodent models of dWMI, effectiveness of MSC therapy has been explored in larger animal models. Multiple research groups have set up preterm sheep models to study dWMI. These models encompass *in utero* surgery between 95 and 102 days of gestation (for human gestation equivalence, see Figure 1) with either transient umbilical cord occlusion (Jellema et al., 2013; Li et al., 2016, 2018) or intra-uterine LPS infusion (Paton et al., 2018) leading to myelination deficits and pronounced OL cell death in the fetal sheep. A pioneer study by Jellema et al. (2013) showed reduction in OL loss, demyelination and microgliosis on histological and MRI-DTI outcome following intravenous human BM-MSC therapy in the fetal lamb *in utero*, 1 h after umbilical cord occlusion. Moreover, MSC administration was shown to affect the peripheral immune response by inducing T-cell tolerance. In a similar sheep model, Li et al. (2016) found an increase in OL numbers, myelin density and decrease of microglia activation and cell death when allogenic ovine UCBCs were administered intra-uterine intravenously at 12 h following transient umbilical cord occlusion to the fetal lamb. Important to note is that UCBCs include MSCs, but also contain lymphocytes, monocytes, and hematopoietic and endothelial stem cells. Interestingly, the therapeutic window proved to be limited. UCBC administration at 12 h after umbilical cord occlusion was effective, however, UCBC treatment at 5 days after the insult was no longer effective (Li et al., 2016). Follow-up studies in the preterm sheep model using intravenous administration of allogenic ovine UCB-MSCs to the fetal lamb *in utero* at 12 h after the insult showed similar results of reduced demyelination through modulation of peripheral and cerebral inflammatory processes (Li et al., 2018). An innovative study by Paton et al. (2018) modeled dWMI in preterm sheep by inducing *in utero* inflammation, a key hallmark of dWMI pathophysiology. Inflammation was induced by intravenous LPS (150 ng) infusion to the fetal sheep *in utero*, during three consecutive days at 95 days (65%) of gestation. 6 h following the final LPS dose, fetal sheep were treated with intravenous human UCBC therapy. UCBC treatment was shown to reduce cerebral gliosis, neutrophil recruitment to the brain and apoptosis, and to restore total and mature OL numbers in the preterm sheep. A recent follow-up study, adopting a similar experimental setup, compared the potential of intravenous human UCBC and human WJ-MSC treatment. WJ-MSCs were shown to be superior in dampening of the (neuro)inflammatory response, defined by lower IL-1β concentrations in the CSF and reduction of glial fibrillary acidic protein (GFAP) coverage in the white matter. However, only UCBCs were capable of reducing OL apoptosis, as measured by an decrease in active caspase-3 staining and a higher number of mature MBP-positive cells. In depth analyses showed a reduction of insulin-like growth factor-1 (IGF-1) expression in the white matter of MSC-treated animals compared to UCBC-treated animals. The authors suggest that enhanced downregulation of IGF-1, a vital growth factor in OL lineage survival and development, could in part be responsible for the absent neuroprotective properties of the WJ-MSCs compared to UCBCs (Paton et al., 2019). All in all this study shows that the working mechanism, the secretome assembly and the response to the local environment of different stem cells (or cellular compositions when using UCBCs) might vary extensively thereby affecting the potency of the different stem cell paradigms. Whether variable beneficial effects of WJ-MSCs versus UCBCs will be found in other experimental models of WMI remains to be studied. Importantly, this study illustrates that to gain optimal neuroprotective or neuroregenerative effects of stem cell therapy it will be crucial to target both neuroinflammation and OL differentiation.

## Strategies to Optimize MSC Therapy in Preterm WMI

The evidence supporting the efficacy and safety of MSC treatment in white matter pathologies is slowly mounting. However, these studies use a wide variety of methodological approaches, varying in important characteristics such as the MSC source, mode of administration and treatment timing. Moreover, efforts to optimize MSC efficacy through cell modification or preconditioning have been made over the years. These important different approaches and various optimization strategies will be discussed below.

### The Source of MSCs

The most optimal source to harvest MSCs is still unclear. While the majority of the early studies use BM-MSCs, the use of MSCs from other sources, such as AD-MSCs and MSCs derived from cord blood or Wharton’s jelly has increased in recent years. BM- and AD-MSCs can be obtained from animals or humans of any age, with cell harvest from adipose tissue being the least invasive. However, studies on the effectiveness of AD-MSCs seem inconclusive. Even though some studies report positive findings of (intravenous or intracranial) AD-MSC treatment on white matter regeneration in animal models of adult stroke and neonatal HIE (Gutierrez-Fernandez et al., 2013a; Park et al., 2013; Otero-Ortega et al., 2015), a very recent study by Sugiyama et al. (2018) has raised some concerns. These authors compared therapy with intravenous rat BM-MSCs versus rat AD-MSCs in a P7 rat model of HIE. Whereas apoptosis and microgliosis were both attenuated in animals treated with BM-MSCs, AD-MSC therapy was not associated with any neuroprotective effects. Importantly, AD-MSC therapy was related to a higher rate of pulmonary complications and mortality.

Although BM- and AD-MSCs can be collected during the whole lifespan, there is evidence linking advanced age to inferior therapeutic potential of the cells (Stolzing et al., 2008; Scruggs et al., 2013; Kalaszczynska and Ferdyn, 2015). Young, undamaged stem cells from the umbilical cord can be obtained without any invasive procedures, and are believed to have higher proliferative potential compared to BM- or AD-MSCs (Park et al., 2018). Even though autologous UCB- or WJ-MSC harvest and culture might not (always) be feasible due to limitations in time, logistics or lead to high variability due to differences in the patient’s clinical condition (for example low pH following birth asphyxia), allogenic UC-MSC therapy is thought to be equally safe (El Omar et al., 2014) and could lead to an off-the-shelf cellular therapeutic strategy with reduced variability in stem cells between patients. WJ-MSCs might be most suitable for allogenic treatment as they are thought to be least immunogenic (El Omar et al., 2014). Moreover, while isolation of MSCs from cord blood was shown to have a low yield of cells, Wharton’s jelly is shown to produce consistent, high yields of MSCs (Zeddou et al., 2010). Recent studies have investigated potential differences in MSC potency as a result of developmental age and maternal conditions in stem cells harvested from the umbilical cord of preterm versus healthy term born neonates (Li et al., 2017; Oppliger et al., 2017). Oppliger et al. (2017) studied *in vitro* neural progenitor cells (NPC) differentiation toward the OL lineage following co-culture with WJ-MSCs, either from a preterm- or term neonatal donor. These authors show that both WJ-MSCs derived from preterm and term deliveries were able to stimulate differentiation of NPCs toward the glial lineage. However, the stem cells differed in their potential to produce mature OLs, as only WJ-MSCs from term deliveries increased the expression of MBP *in vitro*. WJ-MSCs from preterm deliveries did induce an increase of GalC, an immature OL marker, but did not result in maturation of OLs. In line with the study by Oppliger et al. (2017), a recent *in vivo* study by Li et al. (2017) found differences in mode of action between preterm and term UCB-MSCs. In their fetal sheep model both term- and preterm intravenous ovine UCB-MSC therapy reduced preterm WMI by reducing OL cell death, myelin loss and microgliosis. Interestingly, the secondary mechanisms underlying this neuroprotective effect seemed to differ between the cell types. Whereas both preterm and term UCB-MSCs attenuated neuroinflammation, preterm UCB-MSC treatment led to a decrease of TNF-α, while term UCB-MSC therapy caused an increase in anti-inflammatory IL-10. Moreover, term UCB-MSC treatment led to a reduction of oxidative stress, measured by fetal malondiadehyde (MDA) plasma levels, while preterm UCB-MSC treatment did not influence MDA levels. Thus, based on the non-invasive nature of cell harvest, high proliferative potential and apparent superior capacity to produce fully differentiated OLs, we suggest that term WJ-MSCs will perhaps be the stem cell of choice for the treatment of dWMI.

### Route of MSC Administration

Besides questioning the optimal source of MSCs, the most efficient route of MSC administration is also still up for debate. Early studies focused mainly on local intracranial methods of stem cell delivery. Even though intracerebral administration ensures direct and targeted delivery and a minimum loss of stem cells, it is an invasive procedure. In order to avoid intracerebral injections, multiple studies looked at systemic MSC administration routes: either intravenous or intra-arterial applications. Despite being the more convenient and less invasive option, intravenous MSC administration can lead to entrapment of cells in other organs, such as the spleen, kidney, liver, or lungs, leading to a large reduction of cell numbers delivered to the brain (Danielyan et al., 2009). Although cell delivery to the brain is impaired, one could speculate that peripherally lost MSCs could still benefit the preterm patient with multi-organ dysfunction, by possibly dampening peripheral inflammation in the gut or lungs. Moreover, entrapment of MSCs in the spleen or liver has been reported to suppress T-cell activation and to contribute to the inactivation of destructive peripheral immune responses (Kurtz, 2008; Jellema et al., 2013). While intra-arterial MSC injection leads to a higher number of cells in the brain than intravenous application, it can lead to harmful microvascular occlusions (van Velthoven et al., 2010b; Park et al., 2018; Sarmah et al., 2018; Zhang et al., 2018). Interestingly, although this review provides a large body of evidence reporting a beneficial effect of intravenous MSC therapy, a recent meta-analysis including 64 studies regarding adult ischemic stroke found that the effect size and thus therapeutic potential of the invasive intracerebral route was superior compared to other routes of administration (Sarmah et al., 2018). A similar conclusion was drawn by Park et al. (2018), who argued in favor of local delivery of stem cells as delivery in the direct microenvironment of the lesion enhanced the paracrine potential of stem cells. In contrast, a study by Zhang et al. (2018) comparing intracerebral, intravenous and intra-arterial rat BM-MSC therapy in an adult rat model of ischemic stroke reported superior functional recovery, synaptogenesis, neurogenesis and axonal remodeling following intra-arterial MSC delivery compared to the other two administration methods. However, when considering the most optimal route of administration of stem cells for a specific neurological condition, it is vital to take both the pathophysiology of the injury and the clinical condition of the patients into account. In dWMI, OL development is disrupted throughout the developing brain. Even though predilection sites exist in the preterm brain due to spatial and temporal patterns in OL development, the injury, as the term suggests, is diffuse (van Tilborg et al., 2018). Therefore, local delivery of stem cells would be challenging in dWMI, as lesions are spread throughout the brain. Moreover, since extreme preterm infants admitted to the NICU suffer from multiple serious, often life-threatening morbidities, invasive intracranial procedures to deliver stem cells would not be preferable in an unstable patient. More recently, focus has shifted on intranasal MSC administration: a method of cell delivery that is non-invasive, direct, rapid, safe, and which evades loss of cells in the periphery (Danielyan et al., 2009). The possible migration routes following intranasal MSC administration cells were nicely illustrated by Danielyan et al. (2009). In short, stem cells are thought to pass the cribriform plate and migrate toward the lesion site through the olfactory bulb and brain parenchyma, CSF, trigeminal nerve and meningeal circulation. Experimental studies in models of SAH, MS, neonatal HIE and dWMI all show a beneficial outcome following intranasal MSC therapy, promoting endogenous repair of the brain (van Velthoven et al., 2010b; Donega et al., 2015; Oppliger et al., 2016; Nijboer et al., 2018). When comparing the effectiveness of the intranasal route to the intracerebral route of mouse BM-MSC delivery in a P9 mouse model of neonatal HIE, van Velthoven et al. (2012a) reported very similar functional recovery in mice with HIE-related injury. It is important to note that the treatment window in both intranasal as well as systemic administration of MSCs is most likely limited, due to loss of chemotactic signaling and recovery of BBB integrity, complicating MSC migration (Nessler et al., 2013; Donega et al., 2014a). Apart from a limited time window for cell migration, the optimal window of MSC efficacy in dWMI is still up for debate. Preclinical studies in adult ischemic stroke, severe IVH and neonatal stroke demonstrated superior therapeutic efficacy in early (<48 h after injury induction) versus late (>7 days after injury induction) MSC treatment (Kim et al., 2012; Wang et al., 2014; Park et al., 2016). Interestingly, van Velthoven et al. (2010a) reported a treatment window of at least 10 days following injury induction in the P9 HIE mouse model. However, while early treatment could potentially give superior efficacy, pinpointing the exact timeframe in which injury develops and thereby determining the optimal treatment timing in the preterm infant is challenging. Namely, preterm infants encounter various potentially damaging insults consecutively, and multiple insults increase the risk of myelination failure (Rezaie and Dean, 2002; Zhao et al., 2013). Currently, dWMI diagnosis is based on MRI around term-equivalent age, when myelination is advancing (de Vries et al., 2013). In recent years, identification of biomarkers to predict neonatal brain injury has received increasing attention. In these studies multiple biomarkers in the blood, such as S100B, GFAP and metabolites as well as non-invasive monitoring such as EEG and NIRS have been shown to predict HIE, IVH, PHVD, and PVL (Douglas-Escobar and Weiss, 2012; Stewart et al., 2013; Jin et al., 2015; Lee, 2017). However, biomarkers for early identification of dWMI are still lacking. Future research is needed to identify the population of preterm infants that will develop dWMI, ensuring timely treatment to prevent myelination failure.

### Optimizing the MSC Secretome

Other efforts in order to optimize MSC therapy are being made by targeting the paracrine potential of MSCs. Methods aiming to boost the MSC secretome can roughly be subdivided in two approaches, (1) preconditioning of MSCs, and (2) MSC modification. The first approach aims to optimize MSC paracrine functioning by subjecting the cells to an “adverse” event *in vitro*. These events are believed to prime the stem cells, making them more responsive and efficient upon arrival at the lesion site (Cunningham et al., 2018). Some of these preconditioning studies aim at enhancing the anti-inflammatory potential of MSCs by priming the cells with pro-inflammatory cytokines such as IFN-y and IL-1. In a recent study by Redondo-Castro et al. (2017), a short (5 min) preconditioning period of human BM-MSCs with IL-1 (both α and β) enhanced the anti-inflammatory potential of the cells as a result of increased granulocyte colony stimulation factor (G-CSF) production, leading to a reduction of pro-inflammatory IL-6 and TNF-α production by cultured mouse microglia. In contrast, priming of human BM-MSCs with TNF-α or IFN-γ did not enhance the MSC potential (Redondo-Castro et al., 2017). In contrast, Morioka et al. (2017) did report a beneficial effect of IFN-γ pretreatment of human WJ-MSCs. In their P4 rat model of cystic PVL (please see above for description of the model), 4-day i.p. treatment with supernatant of WJ-MSCs pre-treated with IFN-γ did result in an significant increase in MBP-positive area in the brain, while treatment with medium of MSCs that were not preconditioned did not display this regenerative potential. Importantly, IFN-γ was absent in the preconditioned MSC medium, while anti-inflammatory and immunomodulatory factors, namely human tumor necrosis factor-stimulated gene-6 (TSG-6) and indoleamine 2,3-dioxygenase (IDO) were increased. Apart from preconditioning of MSCs using inflammatory stimuli, hypoxic preconditioning of MSCs has been proposed to boost the cell migration and survival capacity of MSCs. A study in a mouse model of adult stroke (MCAO) demonstrated a superior effect in migratory capacity of MSCs, as well as functional recovery of the animals following intranasal hypoxic-preconditioned (HP) rat BM-MSC treatment compared to treatment with MSCs cultured under normoxic conditions. Both cell types were equally effective in preventing apoptosis (Wei et al., 2013). Follow-up studies using ICH and neonatal stroke mouse models confirmed the enhanced potential of HP-BM-MSCs for neuronal regeneration and cell homing (Sun et al., 2015; Wei et al., 2015). Another interesting strategy is to precondition MSCs using ischemic brain extracts. Chen et al. (2002) exposed human MSCs (of unknown origin) to brain protein extracts of either stroke (MCAO) rats or control animals and demonstrated changes in the MSCs’ secretome between the conditions. MSCs exposed to ischemic brain extracts showed increased secretion of trophic factors including BDNF, nerve growth factor (NGF), vascular endothelial growth factor (VEGF), and hepatocyte growth factor (HGF). While this method is maybe not the most applicable option for the clinic, triggering the MSCs and thereby boosting their secretome prior to cell administration could possibly have added beneficial effects, though this needs to be examined in future preclinical studies. Other preconditioning methods, though studied less intensively in (neonatal) WMI, include serum- or medium-preconditioning or priming of MSCs with melatonin, respectively leading to increased MSC survival and functioning, and cell proliferation in the ischemic micro-environment following ischemic brain injury (Tang et al., 2014; Kim et al., 2016).

A different method to boost the MSC secretome is inducing overexpression of (trophic or immunomodulatory) factors by means of genetic engineering. The beneficial effect of modified MSCs has been studied in multiple brain pathologies. A number of preclinical adult MCAO studies found enhancement of functional recovery and reduction of infarct size following treatment with modified BM-MSCs, either overexpressing BDNF, glial cell line-derived neurotrophic factor (GDNF), hypoxia-inducible factor 1a (Hif-1a) or IL-10 compared to treatment with naïve BM-MSCs (Kurozumi et al., 2005; Lv et al., 2017; Nakajima et al., 2017). Other studies, more focused on white matter regeneration following modified MSC therapy, find a similar superior treatment efficacy of modified MSCs versus naïve MSCs. For instance, Liu et al. (2010) showed enhanced reduction of myelin loss, measured by luxol fast staining, in a rat model of adult hemorrhagic stroke, following intracranial treatment with human WJ-MSCs overexpressing HGF compared to animals receiving naïve MSCs. In a cuprizone mouse model of MS, intracerebrally administered IL-13-overexpressing mouse BM-MSCs were shown to superiorly attenuate microgliosis, OL apoptosis and demyelination when compared to naïve BM-MSCs (Le Blon et al., 2016). Another study in the MS field, using an EAE mouse model reported improved functional recovery, greater reduction of pro-inflammatory cytokines in peripheral blood and enhanced reduction of cleaved caspase 3-positive (i.e., apoptotic) cells following intracerebral treatment with human ciliary neurotrophic factor (CNTF)-overexpressing MSCs versus naïve MSCs (origin unknown) (Lu et al., 2009). A study performed in our center in a mouse model of neonatal HIE found that intranasal treatment with mouse BDNF- or sonic hedgehog-overexpressing BM-MSCs led to additional reduction of MBP area loss when compared to naïve MSCs or vehicle-treated animals (van Velthoven et al., 2014). Although this strategy of genetic engineering seems very promising, it is associated with some safety concerns. Viral integration in the MSCs’ genome might boost tumorigenicity. For that reason the use of adenoviruses to deliver the gene of interest into the MSCs could be preferable, as these viruses do not integrate into the hosts DNA (Schäfer et al., 2016; Park et al., 2018). Even though caution is advised, a clinical phase 1/2a trial for patients with adult stroke studying intracranial application of Notch-1-transfected human BM-MSCs reported no safety concerns, in addition to a favorable outcome at 12 months post-treatment (Steinberg et al., 2016).

### Cell-Free Approaches: Stem-Cell Conditioned Medium and Extracellular Vesicles

Additional support for the vital role of the MSC’s secretome comes from studies using either MSC-CM, or extracellular vesicles (EVs) released by MSCs in the treatment of brain injury. CM is defined as medium in which MSCs are cultured during variable lengths of time before collection and is thought to contain all elements of the MSC secretome, both paracrine secreted trophic and anti-inflammatory factors plus EVs (Cunningham et al., 2018). MSCs are believed to secrete multiple types of EVs, including exosomes and microvesicles, which arise from the endosomal compartment and from the plasma membrane, respectively. Both types of EVs contain a range of different cargos, such as mitochondria, messenger RNA (mRNA) and regulatory microRNA (miRNA), cytokines, and other proteins.

#### Conditioned Medium

Jadasz et al. (2013) compared the effect of unconditioned medium versus rat BM-MSC-CM (conditioned during 72 h) on the differentiation potential of primary cultured rat OPCs. Exposure to CM resulted in a boost of OPC maturation compared to unconditioned medium, measured by upregulation of myelin expression, increased MBP protein levels and immunopositive staining, and downregulation of important inhibitory signals. Similarly, when exposed to rat BM-MSC-CM conditioned during 72 h, rat NSCs differentiated toward the oligodendroglial lineage, shown by an increased MBP and CNPase gene expression compared to standard NSC medium, even when NSCs were challenged with growth factor withdrawal or were exposed to an astrogenic stimulus (Steffenhagen et al., 2012). A few *in vivo* studies also report a positive effect of CM therapy in experimental models of WMI. Bai et al. (2012) showed an increase in functional recovery and a reduction in demyelination, as measured by luxol fast blue staining, following intravenous human BM-MSC-CM treatment in a mouse model of autoimmune EAE. By studying the protein content of the CM, the authors discovered an important role of HGF in recovery of myelination. Exogenous intravenous HGF treatment resulted in recovery of myelination as well, while antibodies aimed against HGF or its receptor blocked the regenerative effects of both HGF and CM treatment. However, another study compared intracerebral rat BM-MSCs injections together with MSC-CM (conditioned during 24 h) injections into the lesion, and demonstrated a superior effect of live MSCs on the regeneration of the white matter in a rat model of cPVL when compared to CM only (Chen et al., 2010). The latter study indicates that continuous trophic factor or vesicle production by using actual MSCs, harboring a “regenerative niche” during several days, might be vital for optimal white matter regeneration.

#### Extracellular Vesicles

More recently, studies have focused on the use of EVs for white matter repair. A recent review by Cunningham et al. (2018) provides an excellent overview of the use of EVs in preclinical MCAO models, reporting positive effects of EVs on WM regeneration following adult stroke. In one of these studies, the authors reported white matter repair in adult rat subcortical stroke model as a result of a single intravenous administration of rat AD-MSC-EVs. The EV infusion led to an increase in expression of both CNPase and myelin oligodendrocyte glycoprotein (MOG), which are (early) mature OL markers, restored axonal myelination and improved mean axial diffusivity on DTI compared to vehicle-treated controls (Otero-Ortega et al., 2017). Moreover, another study using a mouse model of progressive MS (i.e., Theiler’s murine encephalomyelitis virus (TMEV)-induced demyelinating disease) demonstrated that intravenously administered human AD-MSC-EVs were capable of improving functional outcome, attenuated neuroinflammation and boosted myelin expression in the mouse brain (Laso-Garcia et al., 2018). Similar findings have been reported in the field of preterm WMI. A pioneer study by Ophelders et al. (2016) using an ovine model of preterm WMI showed a reduction in seizure activity and partial protection against HI-induced myelination deficits following intravenous human BM-MSC-EVs therapy. However, EV treatment did not reduce OL apoptosis or cerebral inflammation. The authors suggest that while MSCs are able to “sense” the micro-environment and polarize toward an anti-inflammatory phenotype, EVs are static and therefore might lack immunomodulatory capabilities (Ophelders et al., 2016). A recent study by Drommelschmidt et al. (2017) did report reduction of gliosis following intraperitoneal human BM-MSC-EVs in an inflammatory model of preterm WMI (0.25 mg/kg LPS in P3 Wistar rats). Aside from reducing neuroinflammation, EV therapy reduced hypomyelination measured with MBP staining, and restored FA values up to SHAM control levels measured by DTI. Even though these studies all report (partial) neuroprotective of regenerative effects of MSC-EV therapy, a direct comparison between MSC and MSC-EV therapy in dWMI has not been made. To the best of our current knowledge, only one study directly compared the efficacy of MSC-EV and MSC therapy directly in a mouse model of brain injury. Doeppner et al. (2015) reported comparable therapeutic effects of human BM-MSC treatment and human BM-MSC-EV in an adult MCAO mouse model. Both therapies potently promoted functional recovery and neurogenesis following stroke induction. It is, however, noteworthy that EV therapy failed to reduce cerebral immune cell infiltration whereas MSC therapy was capable of reducing leukocyte influx (Doeppner et al., 2015). Thus, both these authors and Ophelders et al. (2016) provide evidence for a more potent anti-inflammatory response of MSC therapy compared to EV treatment. The exosome content responsible for the observed regenerative effects is yet to be elucidated. An interesting *in vitro* study by Xiao et al. (2018) suggests an important role for a specific miRNA, miR-134. This miRNA was, among 8 other candidates, found in rat BM-MSC exosomes and has been shown to inhibit OL apoptosis in a primary rat OPC culture after oxygen and glucose deprivation, by targeting caspase 8 (Xiao et al., 2018).

**FIGURE 2:**
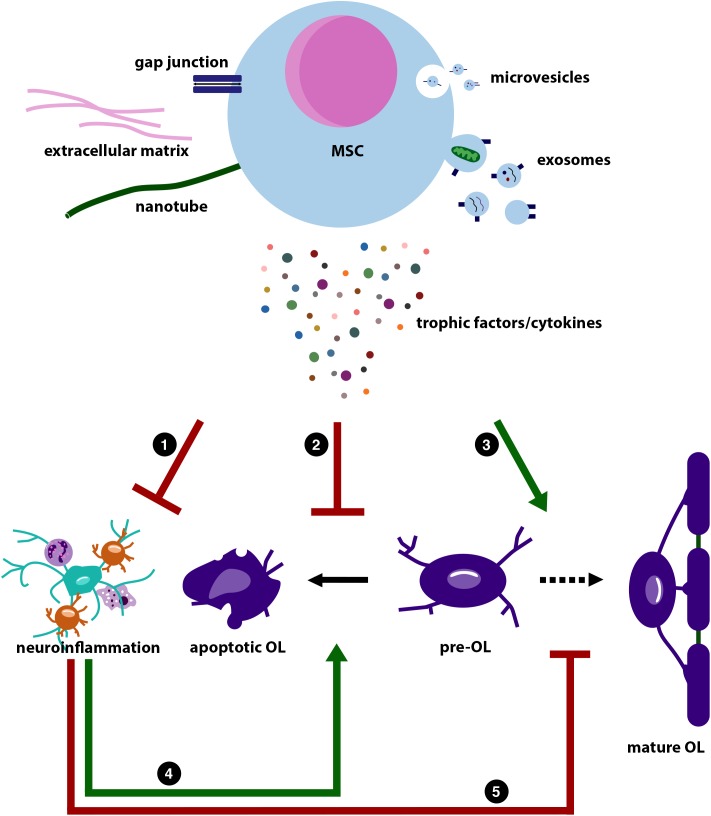
Illustration summarizing the possible working mechanisms of mesenchymal stem cells (MSCs). This illustration is based on the literature discussed in this review. MSCs are believed to exert their regenerative potential through reciprocal transport via gap junctions (blue), production of extracellular matrix proteins (pink), such as laminin, and transport via nanotubes (green). In addition, MSCs are able to release microvesicles (top, small vesicles) and exosomes (bottom, larger vesicles). These vesicles contain a mix of miRNA, cytokines and trophic factors, and mitochondria. MSCs can also directly have paracrine effects on neighboring cells by release of trophic factors and cytokines. Direct cell contact and the MSCs’ secretome can (1) dampen (red inhibitory arrow) the immune response in the periphery and central nervous system (CNS) (astrocyte in green; microglia in orange; neutrophil left top; and macrophage right bottom). The MSCs’ secretome can also (2) directly inhibit apoptosis of pre-myelinating OL (pre-OL; irregular shaped purple cell), whereas it can also (3) directly stimulate (green stimulatory arrow) pre-OL differentiation toward myelinating mature OLs (middle and right purple cells). As neuroinflammation will (4) increase pre-OL apoptosis, and (5) inhibit pre-OL maturation [lower stimulatory arrow (green) and lower inhibitory arrow (red), respectively], MSCs can also indirectly reduce pre-OL apoptosis and stimulate pre-OL maturation by dampening neuroinflammation.

In conclusion, CM or EVs seem potent alternatives to whole MSCs to restore myelination deficits in multiple animals models of brain injury. For an overview on the possible mechanisms of action of MSCs including cell-free approaches, see Figure 2. The use of these cell-free strategies to treat dWMI could prove to have superior clinical applicability compared to live cell administration, that theoretically could raise some safety concerns. However, solid future research comparing the efficacy of CM- or EV-based treatments to conventional MSC therapy in dWMI is needed.

## Concluding Remarks

Currently, a large body of evidence supports a role for MSCs to protect and restore damage to the white matter of the brain. Studies in the field of adult stroke, MS and multiple neonatal brain pathologies underline the anti-inflammatory, immunomodulatory and trophic properties of MSCs, most likely mediated by the MSC secretome. However, several challenges have to be overcome when translating experimental data of MSC treatment to the preterm dWMI field, and eventually toward clinical application.

Even though evidence supporting the beneficial potential of MSCs in boosting (re)myelination following injury is mounting, the number of preclinical studies supporting the efficacy of MSC therapy in dWMI remains limited. This limited amount of evidence is important to consider, as the pathophysiology underlying preterm dWMI is substantially different from other neonatal or adult brain pathologies. In (neonatal) stroke and HIE, the loss of WM volume is, at least partially, the result of loss of white matter (most likely secondary to gray matter loss) with a pronounced role of OL cell death, while in MS immune system dysfunction leads to demyelination (Compston and Coles, 2002; Gutierrez-Fernandez et al., 2013b; Mifsud et al., 2014). Even in other preterm white matter pathologies, such as IVH and cystic PVL, WM loss is most likely more a result of OL apoptosis rather than impaired OL maturation as proposed in dWMI (Volpe et al., 2011; Buser et al., 2012). Thus, more research is needed on MSC therapy in clinically relevant models of specifically preterm dWMI, preferably in both rodents and larger species.

Another challenge for future clinical application is determining the best MSC treatment strategy. Due to methodological differences in experimental design, including MSC origin and mode of administration the optimal treatment protocol is currently unclear. Based on present literature, intranasally applied term WJ-MSCs might prove to be most optimal candidate, due to non-invasive cell harvest and a clinically applicable non-invasive administration route combined with excellent cell homing and paracrine properties of the cells (Li et al., 2017; Oppliger et al., 2017). However, to substantiate this statement, additional preclinical studies in models of dWMI, with a back-to-back comparison of the efficacy of multiple cell origins and routes of administration are needed.

In this review a number of strategies has been discussed to further optimize MSC therapy, all aimed to promote or adapt the anti-inflammatory and trophic factors within the MSC secretome. These options all seem promising, particularly hypoxic preconditioning and MSC genetic modification, but lack sufficient evidence in the dWMI field at present. More importantly, while CM and EV studies underline the vital role of paracrine signaling in MSC-mediated WMI recovery, the specific beneficial mediators of the MSC secretome remain unclear. Insight in the trophic and immunomodulatory factors, and other regulators (such as miRNA) in the MSC secretome underlying the boost of myelination of the preterm brain would not only provide a good basis for MSC optimization (i.e., overexpression studies) but also pave the way for potential cell-free treatment options, such as a cocktail of preferred beneficial growth factors. Cell-free strategies could be the more clinically desirable option, as these alternatives can be easily stored without any concerns on cell viability or safety. However, when taking into consideration the outcome of current CM and EV studies, it is still questionable whether these alternatives will truly replace the need of a whole cell-based therapy. Based on the evidence provided in this review, a regenerative niche harboring continuous (at least days-long) secretion of trophic factors, or possibly direct cell contact between MSCs and neural progenitors is more desirable than transient treatment with MSC derivatives. Therefore, additional research comparing the efficacy of cell-free (either EV, CM, or, growth factor cocktails) alternatives to whole-cell MSC therapy in dWMI models is urgently needed. Moreover in future, combination therapies of MSCs with other regenerative strategies, such as specific trophic factor supplementation, might prove to even further benefit the injured preterm brain.

Despite the fact that there are still quite some challenges to overcome before optimal clinical translation, this review shows that treatment with MSCs or its derivatives is a near-future favorable and promising novel regenerative treatment strategy to improve the prospects and quality of life for preterm infants suffering from dWMI.

## Author Contributions

JV and MV performed the literature search including the reading of selected literature. JV drafted the manuscript in collaboration with CN. CdT, FH, and MB revised the manuscript.

## Conflict of Interest Statement

The authors declare that the research was conducted in the absence of any commercial or financial relationships that could be construed as a potential conflict of interest.
